# Hospital Architecture

**Published:** 1910-02-19

**Authors:** 


### Hospital Architecture.

We shall be glad to receive suggestions and contributions for this Section.—Ed. "The Hospital."

One of the complaints that the practical man has to make against many modern hospitals is that they are ludicrously ugly and inartistic on the outside. There are, of course, exceptions. Some notable ones, indeed, are too artistic, and have their exteriors of too flamboyant a style to be wholly admired by the practical man. The tendency towards exaggerating the importance of an artistic exterior has been most pronounced in Germany, where the large modern municipal hospitals are veritable palaces from an architectural point of view, with pillared façades and richly decorated gable and roof work. In this country, however, that tendency has not been greatly fostered. With exceptions such as the new infirmaries, the additions to certain provincial hospitals and a few special Metropolitan institutions, such as the new buildings of King's College Hospital, the majority of our hospitals are hardly handsome. Our old institutions that have been created in still older buildings which were formerly real palaces, and in the erection of which the architects paid a proper regard to the beauty of things, compare very favourably with the best modern Continental hospitals, where "architectonic beauty" appears to have been a prime consideration. But most of our remaining hospitals are ugly and ill-looking, if not so much in the front aspect, then at least decidedly in the rear. This is well seen in the case of such institutions as Westminster Hospital, which is fine enough when viewed from Dean's Yard, but hardly a handsome structure from any other point of view.

This is a point which hospital designers should carefully consider. Where it is possible, without going to unjustifiable and unnecessary expense, to add to the architectural beauty of the hospital buildings such addition should be made, for it will be a good investment in every sense of the word. It is wonderful what an alteration the judicious application of rough cast and cement dressing—which are among the cheapest of mural adornments—can make, while equally admirable results may be obtained by using brick or stone facings. At present a great deal of attention is bestowed upon the façade or front entrance of the hospital, while the sides and rear are neglected. This is all very well when the institution exists in the midst of a mass of houses among which it would be difficult to find a bare space from which to view the building. Where, however, the hospital is erected within its own grounds, with acres of clear space around it, there is much less excuse for neglecting the exterior beyond the front entrance. It is of the highest importance to a community that its hospital should impress the beholder, and to impress it should possess beauty as well as dignity. These can only be secured by careful consideration of all points when the hospital is being built upon paper. The architect is responsible for the exterior, not the advisory committee or expert, and it would be well if hospital architects, before venturing to execute a design for a new building, would visit some of the really handsome hospitals and attempt to incorporate some of their beauties in the new designs. The time has long passed when a hospital interior was allowed to resemble a barrack dormitory; it is high time that it should be accounted a disgrace for a hospital to look, from the outside, like a gaol or a manufactory.

